# Tribological Behavior of the 316L Stainless Steel with Heterogeneous Lamella Structure

**DOI:** 10.3390/ma11101839

**Published:** 2018-09-27

**Authors:** Wenbo Qin, Jiajie Kang, Jiansheng Li, Wen Yue, Yaoyao Liu, Dingshun She, Qingzhong Mao, Yusheng Li

**Affiliations:** 1 School of Engineering and Technology, China University of Geosciences (Beijing), Beijing 100083, China; qwb_strive@126.com (W.Q.); yw@cugb.edu.cn (W.Y.); lyy_strive@126.com (Y.L.); shedingshun@163.com (D.S.); 2 Nano and Heterogeneous Materials Center, School of Materials Science and Engineering, Nanjing University of Science and Technology, Nanjing 210094, China; 216116000150@njust.edu.cn

**Keywords:** 316L stainless steel, tribological behavior, heterogeneous lamella structure, adhesive wear, abrasive wear, oxidative wear

## Abstract

In this paper, the tribological behavior of 316L stainless steel with heterogeneous lamella structure (HLS), prepared through 85% cold rolling technology and subsequent annealing treatment (750 °C, 10 min), were conducted on a ball-on-disc tribometer under different normal loads in dry ambient air conditions. The morphologies, structures, and compositions of the raw and worn surfaces were analyzed by 3D surface profilometer, XRD, SEM, EDS and TEM. Based on this, the results showed that the HLS 316L stainless steel samples exhibited lower and more steady friction coefficients than coarse-grained samples, especially under higher loads, which can be attributed to the existence of numerous oxidative particles across sliding interfaces. However, the wear resistance of HLS 316L stainless steel sample was a little weakened compared to that of the coarse-grained sample under a normal load of 5 N. When the load increases up to 15 N, an obviously decreased wear resistance was found for the HLS of the 316L stainless steel sample, which was 50% lower than that of coarse-grained sample. This can be ascribed to the more severe oxidative and abrasive wear performance of HLS 316L stainless steel sample under dry sliding conditions.

## 1. Introduction

The 316L austenitic stainless steels have been wildly used in engineering applications as work piece materials, due to their excellent properties, such as corrosion resistance, good formability, and high work-hardening capacity [1,2,3]. In a boundary lubrication regime, for work pieces with relative motion, it cannot be guaranteed that the sliding surfaces are always in well lubricated conditions. Hence, at certain moments, the sliding interfaces may directly be subjected to the dry sliding regime. For steel-on-steel sliding contacts, especially under dry sliding conditions, the deformation and microstructure evolution are of great significance to the tribological behavior of a tribosystem. Those evolutions of subsurface microstructure are crucial in determining the material removal processes [4,5,6]. Additionally, the microhardness and microstructure of sliding pairs seriously affect the tribology behavior [7,8,9]. Consequently, it has become urgent to needs systematically understand its intrinsic tribological mechanism, which will greatly be advantage to guide industrial applications. 

Many previous works indicated that the friction coefficients (0.2–1.5) of stainless steels are fluctuant, sliding with other materials [8,9,10,11,12,13,14,15]. This can be ascribed to their adhesive wear characteristics [16,17]. Oxidative wear, adhesive wear, and abrasive wear are the main material removal mechanisms for stainless steels and other alloys [11,17,18,19,20,21]. Those tribological performances were intensively dependent on the normal loads, counterparts, temperature, and lubricant conditions [16,17,22,23,24]. Vereschaka reported that the friction coefficient and adhesion effect change substantially with different high temperature conditions [25]. Fellah et al. [10] confirmed that both the friction coefficient and wear loss of 316L stainless steel were obviously increased with the increase of normal loads in ambient air condition (humidity ~ 38%). Similarly, for eutectoid steel, Mishra et al. [13] reported that the total wear loss increases with the increase of normal loads. They believed that a superior wear resistance is directly correlated to its initial high hardness and the formation of a hard tribolayer during sliding. As described in a classical Achard equation [26,27,28], the wear volume was inversely proportional to the hardness of materials. In ambient air conditions with a relative humidity of 45% [29], or under lubrication with molybdenum dithiocarbamate (MoDTC) and zinc dialkyldithiophosphate (ZDDP) conditions [30], a nanostructure metal with high hardness was frequently reported that it may have the enhanced wear resistance compared with its coarse-grained state [29,30]. However, the Achard equation may be valid only with the existence of dominant physical wear regimes, such as adhesive wear and abrasive wear [26,27,31,32]. When the chemical wear regimes, such as oxidation wear mechanism, controlled the wear loss process, the deterioration in wear resistance could also be revealed for 316L stainless steels. The oxidative products can act as the relatively hard particle across the sliding interfaces, which can also play an important role in increasing material removals via forming plenty of furrows on the worn surface due to the ploughing effect of hard particles [33,34]. Recently, the 316L stainless steel and other alloys with heterogeneous lamella structure (HLS) containing lamellar coarse grains sandwiched between mixtures of nanotwins and nanograins, were constantly reported [35,36,37,38]. These HLS metallic materials possess superior strength and ductility than their coarse-grained state, which have been confirmed to have huge potential for the structural application. However, their tribological performance has not been clearly explored. The introduction of a great deal of nanograins and nanotwins may strongly affect the tribological behavior of HLS metallic materials by the following two points: (1) it results in the increase of grain boundaries, which can adsorb more oxygen species in grain boundaries and, thus, it can accelerate oxidative wear; (2) the enhanced strength and hardness from nanograins and nanotwins may improve wear resistance (Achard’s law). Hence, especially in dry sliding conditions, the interpretation of the tribological behavior of HLS 316L stainless steel is beneficial to lubricant design for work pieces in practical application. 

In the present work, the tribological behavior of HLS 316L stainless steels sliding against GCr15 balls was systematically explored under various normal loads in dry ambient air conditions. Special efforts were devoted to reveal the dominant tribological mechanisms of HLS 316L stainless steel with different microstructures.

## 2. Experimental

### 2.1. Material Preparation and Characteristics

**Figure 1:**
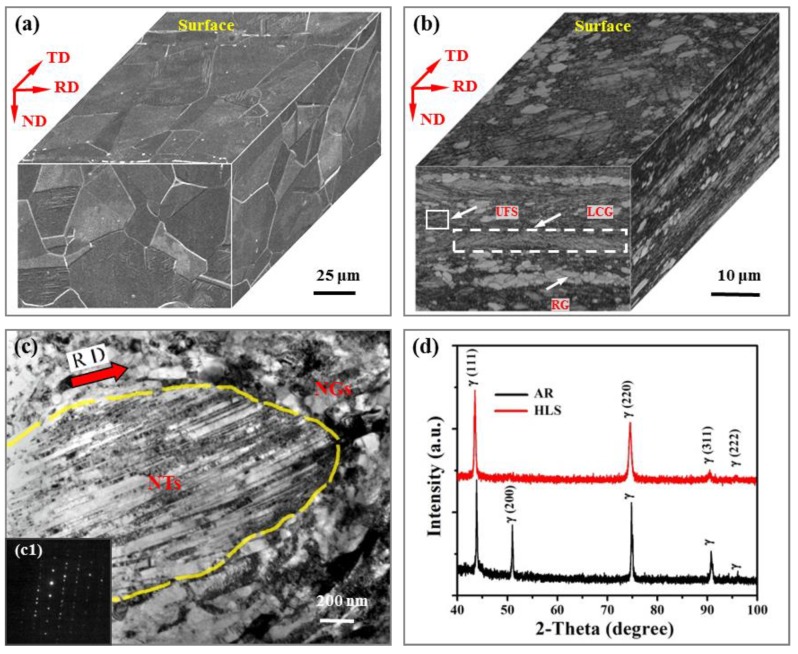
(**a**,**b**) are three-dimensional microstructures of as-received and heterogeneous lamella structure (HLS) 316L stainless steels, the ND, TD, and RD refer to normal direction, transverse direction, and rolling direction, respectively; (**c**) The typical TEM images of ultrafine structure (UFS) in (**b**), which is composed of nanotwin bundles (NTs) and nanograins (NGs); (**d**) XRD pattern of as-received and HLS 316L stainless steels.

The as-received sample used in this present work was a typical 316L austenite stainless steel. Its chemical compositions are shown in Table 1. As it is shown in Figure 1a, the three-dimensional microstructure of as-received sample exhibits approximately equiaxed austenitic grains. Its average grain size is ~35 μm. For 316L stainless steel, a heterogeneous lamella structure (HLS) was prepared via 85% cold rolling and using a subsequent annealing treatment in air (750 °C, 10 min, under nitrogen protection). The sample sheets were rolled from 10 to 1.5 mm thick after 50 passes (the mean thickness reduction was ~0.17 mm per pass). In order to avoid the influence of samples’ thickness on tribological properties, the as-received sample was also cut into a sample having a thickness of 1.5 mm. After cold rolling and annealing treatment, its hardness increased from 177 to 378 Hv. The detailed introductions for preparation, microstructure, and mechanical properties of the HLS have been reported in our previous works [36,37]. As it is described in Figure 1b,c, the HLS was characterized with lamella coarse grain (LCG) and lamella recrystallization grain (RG) clusters, both sandwiched by the ultrafine structures that consisted of nanograins (NGs, grain size of ~90 nm) and nanotwin bundles (NTs, twin/matrix lamellar thickness of ~50 nm). XRD results are shown in Figure 1d, which further indicate that both the as-received and HLS 316L stainless steel samples are only composed of austenitic phases. It is assured that the difference in tribological performance is due to structural differences, and not due to differences in phase composition. The surfaces of 316L stainless steel sample were polished with a roughness (Ra) of ~45 nm. Moreover, the GCr15 balls with 6 mm in diameter were used as counterparts in the following tribotests.

### 2.2. Ball-on-Disc Tribotests

To evaluate the effect of HLS on the tribological performance of 316L stainless steel, tribotests were performed on a CSM tribometer with ball-on-disc rotation mode in dry ambient air at room temperature (relative humidity 7.5%). The normal load was 5, 10, and 15 N at a constant rotation speed of 400 rpm (or 16.75 cm/s). The rotation radius is 4 mm, and the total sliding test durations were 24,000 cycles or 602.9 m. In order to remove any contamination that may have been left from the sample preparation steps, before each tribological test, both 316L stainless steel samples and GCr15 balls were initially ultrasonically cleaned with acetone for 30 min, and then with alcohol for 30 min. In order to ensure the reliability of tribotest data, each tribotest was repeated three times at the same testing conditions. After tribotests, the wear rate (*W_R_*) of samples was estimated according to the following formula [26,35,37]:

(1) $$W_{R}=V/F_{N}\times L,$$

where *V* is wear volume of the 316L stainless steel samples, while *F_N_* is the normal load, and *L* shows the total sliding distance [39,40].

### 2.3. Detailed Analysis Methods

X-ray diffraction (XRD, Bruker-AXS D8 Advance diffractometer, Karlsruhe, Germany) measurements were conducted with Cu Kα radiation to determine the possible phase composition of as-received and HLS samples. The range of 2-theta is from 40° to 100°, with the scanning speed of 6°/min. The surface roughness of 316L stainless steel samples were measured using the white light interferometer (NanoMap-D). The measurements of scanning electron microscope (SEM, Quant 250 FEG, FEI, Hillsboro, OR, USA) EDS mapping were conducted to characterize the evolutions of microstructures and chemical compositions of wear tracks and wear scars. Ultrafine structures were characterized on the analysis of transmission electron microscope (TEM, TECNAI G2 20 LaB6, FEI, Hillsboro, OR, USA) at an accelerated voltage of 200 kV. TEM specimen preparation contains three steps. Firstly, a sample with the thickness of 0.5 mm was cut from the cross-section regions of sample using wire-electrode cutting techniques, and then mechanically grinded down to a thickness ~50 μm. The final thinning was performed by a twin-jet electrochemical polishing process (a solution of 8% per chloric acid + 92% ethanol at 50 V (80 mA) and around −10 °C).

## 3. Results

### 3.1. Friction Behavior

**Figure 2:**
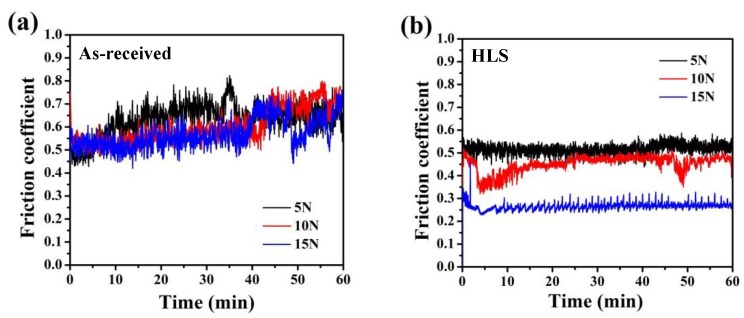
Friction coefficient curves of as-received and HLS samples sliding against GCr15 balls: (**a**) As-received 316L stainless steels under the loads of 5, 10, and 15 N; (**b**) HLS 316L stainless steels under the loads of 5, 10, and 15 N.

Figure 2 presents the friction coefficient curves of as-received and HLS 316L stainless steel samples sliding against GCr15 balls under various loads conditions. The as-received sample exhibits a typical unsteady and fluctuating friction coefficient that ranged from 0.4 to 0.8, which is consistent with many previous works [10,11]. The friction coefficient of as-received 316L stainless steel sample is hardly affected by the normal loads (Figure 2a). However, for HLS samples, with increasing normal loads, the friction coefficients obviously decrease. As shown in Figure 2b, the friction coefficient of HLS sample is relatively more stable and lower than that of the as-received 316L stainless steel sample. The maximum friction coefficient is ~0.52 under the normal load of 5 N, and rapidly decreases to ~0.27 under the load of 15 N. These results are related to the composition and morphologies between tribological interfaces, and the detailed explanation will be discussed in Section 4.

### 3.2. Wear Performance

**Figure 3:**
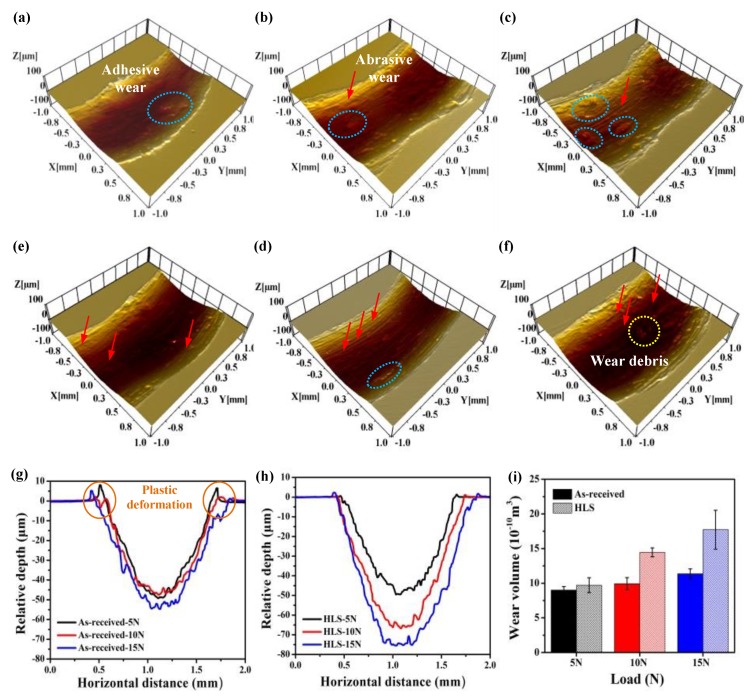
The wear track topographies and wear volumes of the as-received and HLS 316L stainless steels: (**a**) As-received, 5 N; (**b**) As-received, 10 N; (**c**) As-received, 15 N; (**d**) HLS, 5 N; (**e**) HLS, 10 N; (**f**) HLS, 15 N; (**g**) Surface profiles across the wear tracks of as-received 316L stainless steels; (**h**) The two-dimensional cross-sections of wear tracks of HLS 316L stainless steels; (**i**) Wear volumes of the as-received and HLS 316L stainless steels.

Morphologies and wear rates of 316L stainless steels: The wear track topographies and wear rates of the as-received and HLS 316L stainless steel samples are shown in Figure 3. It can be seen from Figure 3a–c that materials easily adhered to the wear tracks of as-received 316L stainless steel samples during sliding, which exhibits a typical phenomenon of adhesive wear. For the HLS 316L stainless steel samples, many furrows with some macroadhesion can be seen in wear tracks (Figure 3e–f). The furrows may be caused by existence of oxide debris in the sliding interfaces, and it can be clearly seen in Figure 3f. Figure 3g–i indicate that the wear rates are positively related to the normal loads, and the wear resistance of HLS 316L stainless steel samples are weaker than those of as-received samples.

### 3.3. SEM and EDS Mapping Measurements on Worn Surfaces

**Figure 4:**
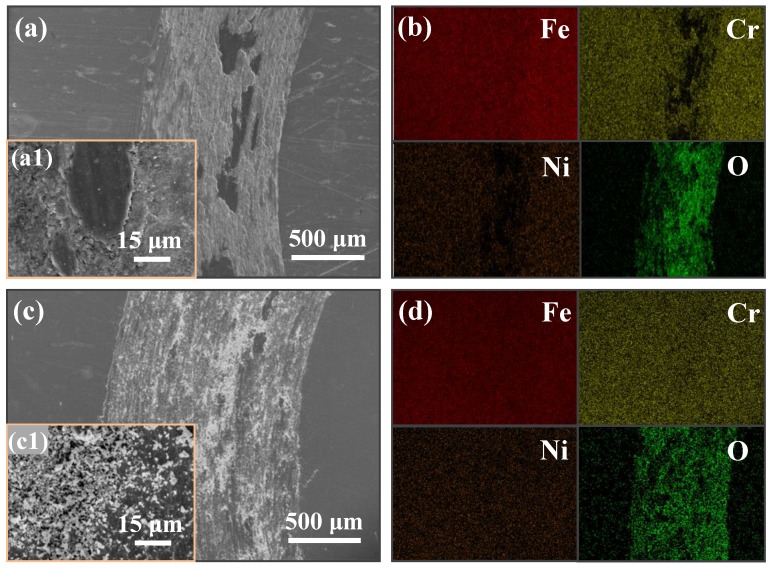
SEM morphologies and the corresponding EDS mapping results of wear tracks of the as-received and HLS 316L stainless steels: (**a**) The wear track of the as-received 316L stainless steel, where the inset (**a1**) is the enlarged morphology of the wear track; (**b**) The corresponding EDS mapping images of (**a**); (**c**) The wear track of the HLS 316L stainless steel, where the inset (**c1**) is the enlarged morphology of the wear track; (**d**) The corresponding EDS mapping images of (**c**).

Morphologies of wear tracks on as-received and HLS 316L stainless steel samples: After tribotests, SEM and EDS mapping measurements were carried out to further understand the characteristics of wear tracks under the normal load of 5 N conditions. Figure 4a shows a typical morphology of adhesive wear phenomenon that counterpart materials are extensively deformed and adhered to the worn surface of wear track. Meanwhile, the inset of Figure 4a shows that the worn surface near to adhesion phenomenon is also covered by some fine debris, which may be confirmed as oxidative debris by Figure 4b. Thus, it may indicate a dominant adhesive wear regime, together with mild tribo-oxidative wear regime for the as-received 316L stainless steel sample under dry sliding conditions. Figure 4c exhibits a distinct wear track morphology of HLS 316L stainless steel samples. It indicates that a lot of oxidized debris, instead of materials adhesion, appeared on the surface of the wear track, indicating a severe tribo-oxidative wear regime.

**Figure 5:**
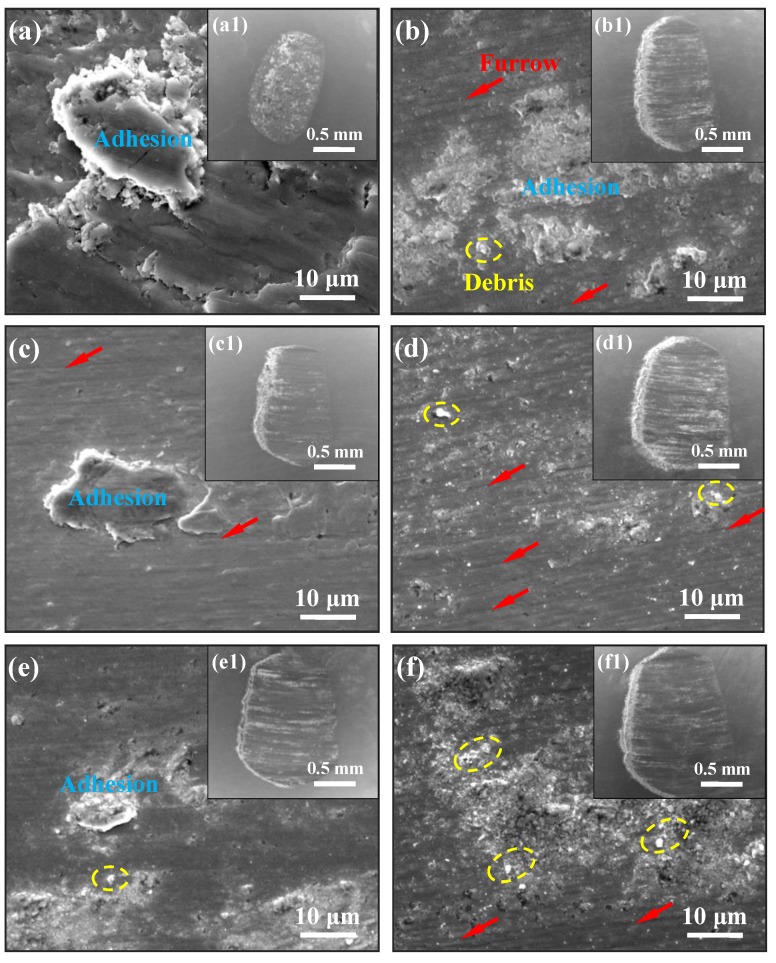
SEM images of the worn surfaces on GCr15 balls under various loads: (**a**,**c**,**e**) show the enlarged morphologies of the wear scars on GCr15 balls sliding against as-received 316L stainless steels with corresponding loads of 5, 10, and 15 N, where the insets (**a1**,**c1**,**e1**) are the whole morphologies of corresponding wear scars; (**b**,**d**,**f**) show the enlarged morphologies of the wear scars on GCr15 balls sliding HLS 316L stainless steels with corresponding loads of 5, 10, and 15 N, where the insets (**b1**,**d1**,**f1**) are the whole morphologies of corresponding wear scars.

Morphologies of wear scars on GCr15 balls: Figure 5 describes the morphologies of wear scars on GCr15 balls. Figure 5a,c,e indicate that the morphologies of wear scar on GCr15 balls sliding against as-received 316L stainless steel samples are mainly composed of material adhesion phenomenon. With the increase of normal loads, the worn surface presents the gradual transition from severe adhesion to medium adhesion phenomena. Meanwhile, more quantity of oxidative debris appeared, which results in many furrows on the worn surface. For the morphologies of wear scar on GCr15 balls sliding against HLS 316L stainless steel samples, they principally consisted of oxidized debris, furrows, and mild adhesion (Figure 5b,d,f). It is observed that the quantity of oxidized debris and furrows increases with the increase of the normal load, and they can act as the third-body abrasive across sliding interfaces, which presents the accelerated tribo-oxidative and abrasive wear behavior.

## 4. Discussion 

**Figure 6:**
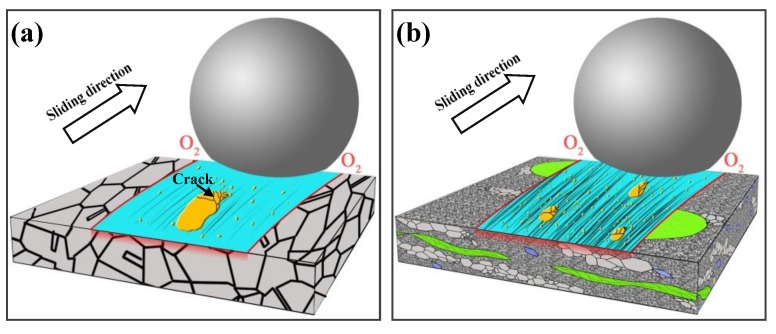
Typical wear mechanisms of as-received and HLS 316L stainless steels sliding against GCr15 balls. (**a**) as-received samples sliding against GCr15, (**b**) HLS samples sliding against GCr15.

**Figure 7:**
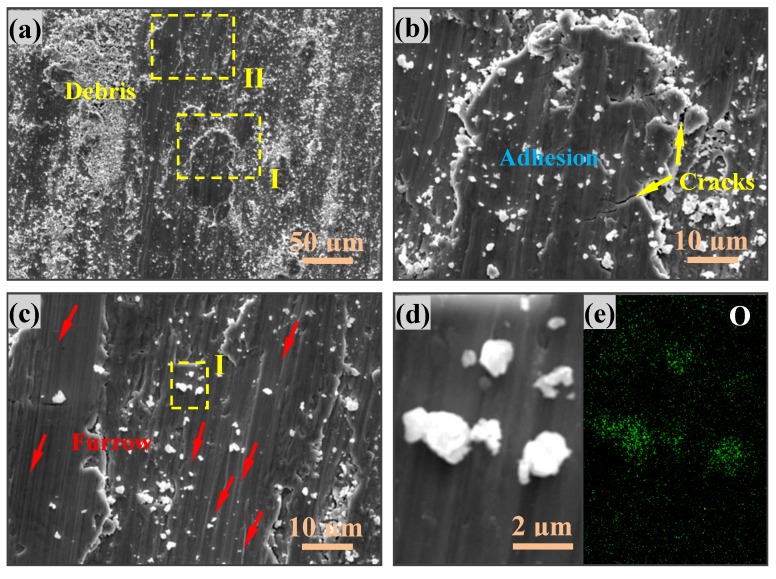
SEM images and EDS mapping images of wear tracks of HLS 316L stainless steels: (**a**) The SEM image of the wear track of HLS 316L stainless steel; (**b**,**c**) are the enlarged images of the selected regions Ι and ΙΙ in (**a**); (**d**,**e**) are the SEM image and corresponding EDS mapping image of the selected region Ι in (**c**).

**Figure 8:**
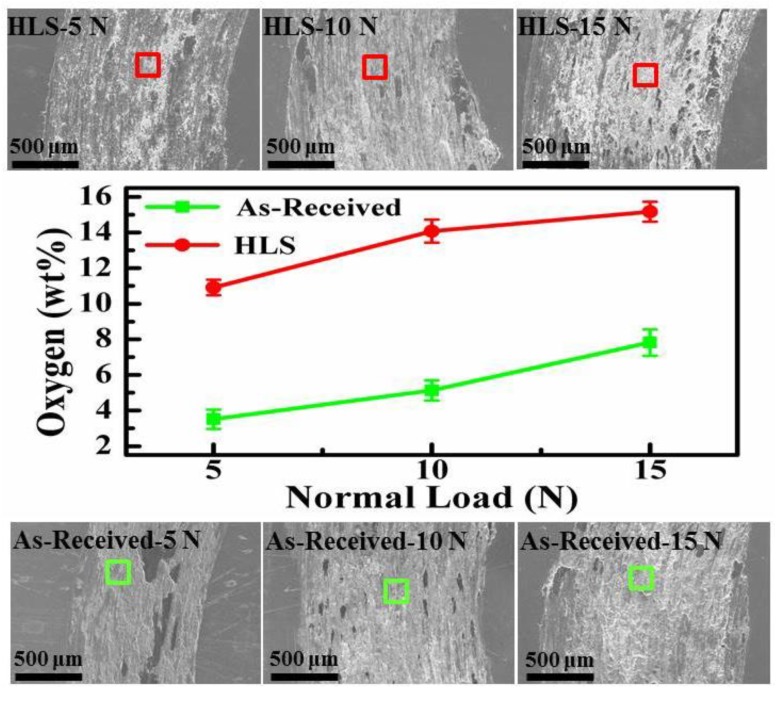
The comparison of oxygen content (wt %) in wear tracks. The red and green boxes represent the testing position of the EDS measurements.

Under dry atmospheric conditions, for the tribological performance of steel, the tribo-oxidation is a key factor in determining the total wear loss [41]. Quinn et al. [18] have reported the oxidative wear mechanism in low alloy steels under various loads and sliding velocity conditions accompanied with the formation of oxidative particles. Due to the complicated material removal process accompanied by tribochemical reaction, there is not just one wear mechanism existing in the sliding interfaces. However, for sliding on steel materials, the adhesive effect inevitably occurs under dry sliding conditions. Figure 6 describes the typical wear mechanisms of as-received and HLS 316L stainless steels sliding against GCr15 balls under dry sliding conditions. It indicates that the wear mechanism transferred from adhesive wear to abrasive wear mechanism with the formation of adhesion, cracks and furrows on worn surfaces. Figure 7 shows the SEM images and EDS mapping images of the wear tracks of HLS 316L stainless steels. Hence, the adhesive, abrasive, and oxidative wear was further confirmed. As it is shown in Figure 6, a large scale of the adhesive phenomena occurred for the as-received 316L stainless steel, while the dispersed adhesive phenomena with small size occurred for HLS 316L stainless steel. As the dry slide continues, tribo-oxidized reactions can ceaselessly take place on the surface of adhesive materials. Under the effect of interfacial shear strength, it may generate a lot of cracks at the edge of the adhesive materials (as it is described in Figure 6 and Figure 7b). Suffering the further extrusion and shear effects, oxidative particles gradually spall from cracks and constantly exist in the sliding interfaces (as shown in Figure 7c). As it is shown in Figure 7d,e, the oxidative particles are continuously crushed at the sliding interface to form particles with a relatively rounded corner. Those oxide particles have a certain antifriction effect at the interface [3]. Meanwhile, previous works have indicated that hard particles with a certain size existing at the sliding interface may significantly decrease friction [33]. In the present work, for HLS 316L stainless steel sample, due to the formation of lamella coarse grain, lamella recrystallization grain cluster, nanograins, and nanotwin bundles, more gain boundaries exist in the HLS samples. As a result, more oxidation species can be absorbed in those HLS. The HLS 316L stainless steel sample is more easily subjected to oxidative wear process. Consequently, the friction coefficients of HLS 316L stainless steel samples decreased compared with the as-received samples. Figure 8 indicates that the different content of oxygen element (wt %) in the wear tracks are strongly related to the microstructures of samples and the normal loads. With the increase of normal loads, the oxygen content increased in wear tracks. Moreover, it still demonstrated that the HLS samples are easily subjected to oxidation effect. The enhancement of the oxidation process markedly increased, accompanied with the thermal effect of the interfaces, and more oxidative particles are formed. Thereby, the friction coefficients gradually decrease, and the wear rates increase. Additionally, oxidative wear debris may serve as the third-body abrasive at the sliding interface, which leads to abrasive wear accompanied with the furrow phenomenon on worn surfaces, due to its ploughing effect. The oxide particles continuously scrapes the exposed material surface of HLS 316L stainless steel, which can result in more material removal. Hence, the conjunct effect of oxidative wear and abrasive wear determines the total wear loss of HLS 316L stainless steel under dry sliding conditions.

## 5. Conclusions

This work presents tribological performances of 316L stainless steels with different microstructures. Additionally, the elucidation on evolutions of tribological mechanisms governing friction and wear behavior is of great significance. Based on the discussion above, the conclusions of this work are as follows.

1. A 316L stainless steel with the heterogeneous lamella structure was successfully prepared through 85% cold rolling and subsequent annealing treatment in air (750 °C, 10 min).
2. The friction coefficient of the heterogeneous lamella structure 316L stainless steel is lower than that of as-received samples with coarse-grained state, which is ascribed to the frictional reduction effect of the oxidative particles generated at the sliding interfaces.
3. The formation of heterogeneous lamella structure is more easily subjected to severe tribo-oxidative and abrasive wear process, which results in the increase of wear rates. Additionally, with increasing normal loads, the enhancement of the oxidation process markedly increases, accompanied with the thermal effect of sliding interfaces, and more oxide particles are formed, and the oxidation wear becomes more severe, accompanied by more wear loss.

## Figures and Tables

**Table 1:** Chemical compositions of 316L stainless steel.

Material	Chemical Compositions (wt %)								
	Cr	Ni	Mo	C	Si	Mn	P	S	Fe
316L	16.47	10.10	1.97	0.03	0.53	1.42	0.03	0.005	Bal.
